# Regulatory Domain Selectivity in the Cell-Type Specific PKN-Dependence of Cell Migration

**DOI:** 10.1371/journal.pone.0021732

**Published:** 2011-07-06

**Authors:** Sylvie Lachmann, Amy Jevons, Manu De Rycker, Adele Casamassima, Simone Radtke, Alejandra Collazos, Peter J. Parker

**Affiliations:** 1 Protein Phosphorylation Laboratory, London Research Institute, London, United Kingdom; 2 Division of Cancer Studies, King's College London, London, United Kingdom; University of Birmingham, United Kingdom

## Abstract

The mammalian protein kinase N (PKN) family of Serine/Threonine kinases comprises three isoforms, which are targets for Rho family GTPases. Small GTPases are major regulators of the cellular cytoskeleton, generating interest in the role(s) of specific PKN isoforms in processes such as cell migration and invasion. It has been reported that PKN3 is required for prostate tumour cell invasion but not PKN1 or 2. Here we employ a cell model, the 5637 bladder tumour cell line where PKN2 is relatively highly expressed, to assess the potential redundancy of these isoforms in migratory responses. It is established that PKN2 has a critical role in the migration and invasion of these cells. Furthermore, using a PKN wild-type and chimera rescue strategy, it is shown that PKN isoforms are not simply redundant in supporting migration, but appear to be linked through isoform specific regulatory domain properties to selective upstream signals. It is concluded that intervention in PKNs may need to be directed at multiple isoforms to be effective in different cell types.

## Introduction

Cell migration is a highly coordinated and tightly regulated process engaging not only cytoskeletal remodelling events at the front and rear of the cell, but also signalling cascades that translate cues in the extracellular environment to determine the polarity of the cell and the direction of movement. Small GTPases of the Rho family are known for their roles in (re)organizing the cytoskeleton (recently reviewed [1]) and in the generation of force important in movement [2]. These actions are exerted through one or more of a number of Rho family effectors (reviewed [3]). These effectors embrace a variety of proteins including scaffolds, cytoskeleton interacting proteins, protein kinases, lipid kinases, phospholipases and oxidases and amongst these is the protein kinase C-related family of serine/threonine protein kinases (PKN1-3).

The PKN family is closely related to the PKC family in their conserved catalytic domains while differing in their N-terminal regulatory domains. The three known isoforms, PKN1, 2 and 3 are closely related, exhibiting greatest variation within their regulatory domains. All 3 isoforms contain three polybasic coiled-coiled motifs the first two of which as elucidated for PKN1 and 2, bind to Rac- and Rho GTPases [4], [5], [6], [7]. Furthermore, the very C-termini of PKN1 and 2 also play a role in binding to RhoA [8], [9]. In addition, PKN1 responds to phosphoinositides [10] and fatty acids such as arachidonic, linoleic and oleic acid [11], [12], [13]; these properties differ for PKN2 [14]. The individual isoforms can be activated upon specific growth factor receptor signalling such as the androgen receptor for PKN1 [15], [16], PDGF and CD44 for PKN2 [17] and insulin for PKN3 [18]. It is therefore not surprising that each isoform has been associated with different adaptor proteins [19], [20], [21], suggesting that PKN isoforms may not functionally substitute for one another, consistent with apparently selective effects of isoforms.

One explanation for the observed PKN isoform specific effects might in part be a consequence of the varying expression levels of the PKNs. While the expression of PKN1 in human tissue ([11], [22]) and PKN2 in mouse tissue [20] is rather ubiquitous, the expression of PKN3 mRNA is relatively restricted to specific tissues including skeletal muscle, heart and liver [22]. PKN3 was found to be upregulated in various tumour cell lines [14], and PKN1 overexpression has been correlated with tumour grade in prostate cancer [16]. PKN3, but not PKN1 or PKN2, was shown to play a role in invasion of a 3D matrix in PC3 cells [18]. This may reflect the relative abundance of PKN3 in PC3 cells or may suggest a unique property of PKN3.

Both PKN1 and 2 can phosphorylate and thereby regulate cytoskeletal substrates. Microinjection of a kinase inactive PKN2 led to the disruption of stress fibres in NIH3T3 cells [4] while kinase inactive forms of PKN1 prevented insulin induced actin stress fibre break down and membrane ruffling [23]. PKN1 interacts with and phosphorylates the microfilament protein α-actinin [24] and PKN2 was reported to phosphorylate cortactin [17]. Cortactin is known to enhance actin polymerization by activating the Arp2/3 complex and hence contribute to tumour cell migration and metastasis [25]. PKN1 can also indirectly influence the contractility of myosin by increasing the inhibitory effects of CPI-17 on myosin phosphatase [26]. Moreover, PKNs have been described to regulate intermediate filaments, for example vimentin glial fibrillary acidic protein as well as neurofilament proteins ([27], [28]). These examples of PKN1 and 2 as possible players in cytoskeleton dynamics together with data that PKN1 can act upstream of MAP kinases JNK [29] and p38 [30], [31] suggest that PKN1 and 2 may be able to drive cell migration in addition to PKN3.

PKN3 has been considered the primary player in PKN-dependent tumour cell invasion [18] with no role for PKN1 or PKN2. Here we have assessed whether this is reflective of expression, function or cellular wiring. We have screened 22 tumour cell lines for their PKN expression signatures. Of these cell lines, we selected 5637 bladder tumour cells as a model for the enriched expression of PKN2. By using siRNA knock-down and rescue, we addressed the contribution of individual PKN isoforms to tumour cell migration and invasion. Our data argue that anti-invasive therapies would be most effective if all 3 PKN isoforms were abrogated.

## Results

### Expression of PKN isoforms in tumour cell lines

**Figure 1:**
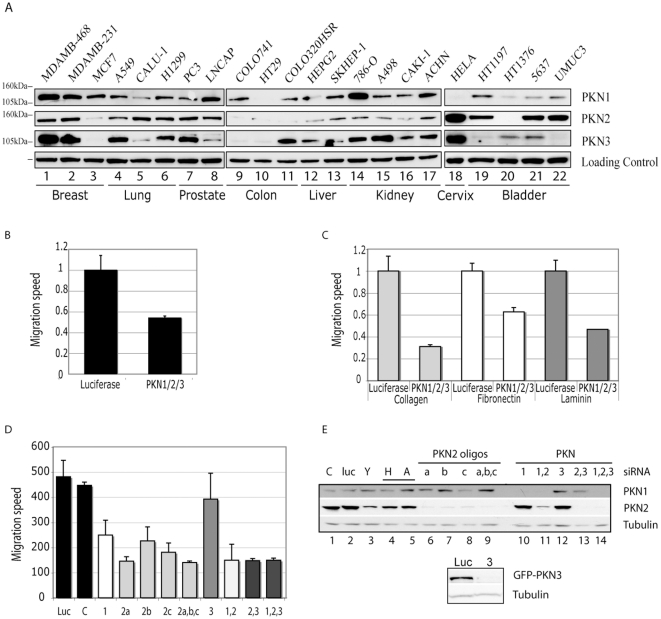
Expression of PKN isoforms in tumour cell lines and effect of PKN isoform knock down on wound healing of 5637 cells. **A.** Protein concentration of cell lysates were analysed by Bradford and aliquots of 30 µg (lanes 1–17) or 50 µg (lanes 18–22) were separated by SDS-PAGE and blotted onto Nitrocellulose. Membranes were probed for PKN1, 2 and 3 (lanes 1–22). Tubulin (lane 1–17) or actin (lane 18–22) served as loading controls. **B.** 5637 cells were grown in uncoated 24well plates and depleted of PKN1, 2 and 3 by triple siRNA transfection. 72 h post transfection confluent monolayers were scratched with a pipette tip and wound healing monitored using a lowlight Microscope and analysed by Metamorph software. siRNA (matched concentration) against Luciferase served as a control. **C.** 24well plates were coated with 10 µg/ml laminin, 25 µg/ml fibronectin or 50 µg/ml collagen for 1 h at 37°C or overnight at 4°C and blocked with 2% BSA for 30 min at 37°C. 5637 cells were then seeded onto the coated multiwell plates, PKN expression was knock-down by siRNA transfection and cells were subjected to a wound healing assay 72 h post transfection as in (B). **D.** 5637 cells grown on 50 µg/ml collagen coated plates, were transfected with PKN isoform specific siRNA. 72 h post transfection wound healing assays were performed on confluent monolayers. Speed of wound closure of luciferase and PKN knock down cells was compared using Metamorph software. Cells were then harvested and analysed by western to assess knock down efficiency (lower panels). **E.** The expression of PKN1 and PKN2 was monitored by western to determine the extent of knock-down under the conditions indicated. For PKN3, fresh batches of antisera (as compared to those employed in panel A) would not detect endogenous PKN3 and knock-down by the siRNA directed at this isoform was confirmed using cDNA expression of a tagged PKN3 (lower panel).

In order to study the contribution of individual PKN isoforms to cell migration we compared 22 tumour cell lines for the relative expression levels of PKN1, 2 and 3. All cell lines expressed at least one PKN isoform, with lines displaying considerable variations (>10-fold) in levels of individual PKNs (Figure 1A). PKN1 was broadly expressed and consistently evident in breast tumour cells; it has recently been identified in an amplicon in triple negative breast cancer [32]. PKN2 protein was also broadly expressed with high levels in 3/4 bladder cell lines. The expression of PKN3 varied the most among tumour cell lines with for example very high and very low levels evident in breast tumour cell lines.

Because of the relative enrichment in the expression of PKN2 in 5637 cells we selected these as the model for analysis by knock-down. In parallel, some studies were also performed in MDA-MB-468 cells where all three isoforms displayed good relative expression. Quantitation of absolute expression employing recombinant PKN proteins to titre antisera, determined that for 5637 cell PKN1, 2 and 3 were expressed at 1.1, 0.21 and 0.08 µg/mg cell protein; for MDA-MB-468 cells these values were: 16.7, 0.12 and 0.44 respectively.

In these model systems, all three PKN isoforms were depleted by siRNA and scratch wound assays were employed to assess requirements for PKN during migration. As shown in Figure 1B for 5637 bladder cells, we observed a ∼50% reduction in speed of wound closure in the absence of PKN1/2/3 compared to the control knock-down cells. The depletion of PKN1/2/3 isoforms in 5637 bladder cells resulted in a significant reduction in the speed of wound closure on all cell matrices tested, but particularly on collagen (Figure 1C). In MDA-MB-468 cells migration was also reduced albeit less robustly and in this model most responsively on fibronectin (Figure S1).

### PKN1 and PKN2 are important for 5637 bladder tumour cell migration

siRNA against individual PKN isoforms or pairwise combinations thereof were tested in wound healing assays on collagen for 5637 cells. Depletion of either PKN1 or PKN2 had a significant effect on migration, while depletion of PKN3 had no effect under any circumstances (Figure 1D). In combination knock-downs for 5637 cells, no greater effect was observed than that of PKN2 knock-down alone and 3/3 PKN2 directed siRNAs were effective in inhibiting migration. Westerns (Figure 1E) indicate the efficacy of each siRNA or combination. For PKN3, variation in batches of purified antibody precluded the routine monitoring of endogenous PKN3, however the efficacy of the siRNA to PKN3 was demonstrated employing a GFP-PKN3 fusion (see Figure 1E lower panel); specificity was evident from the westerns for endogenous PKN1 and PKN2 (Figure 1E upper panel).

### PKN influences speed and direction of movement

**Figure 2:**
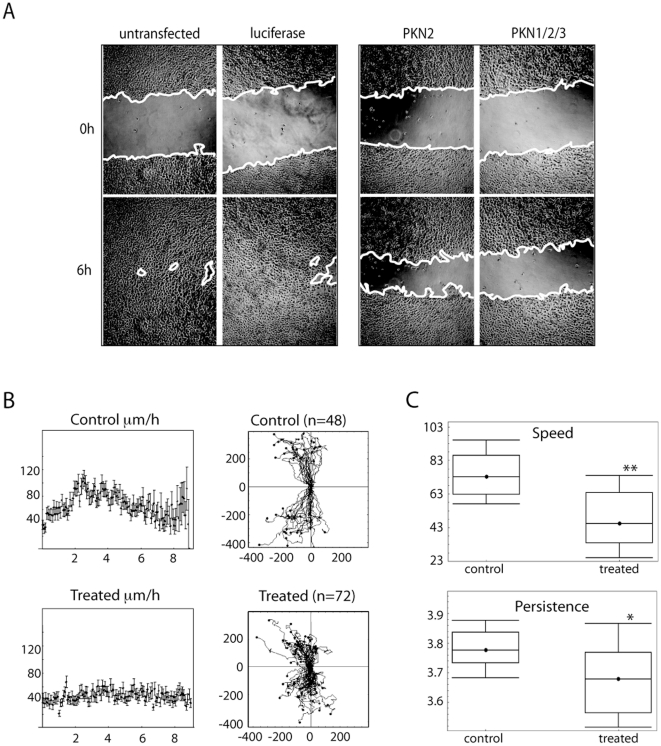
Loss of PKN reduces speed and directionality of 5637 bladder cells during wound healing. 5637 cells grown on collagen coated plates were transfected with siRNA against luciferase, PKN1, PKN2 or PKN3 as indicated. At 3 days post transfection confluent cell monolayers were scratched and wound closure filmed with a lowlight microscope. **A.** Images were taken at the time of wounding (t 0 h) and after 6 h of migration as indicated. **B.** Individual cells were picked at random and tracked during migration using AQM-Tracker software. Graphs show the speed of movement in control and PKN knock down cells over 6 h. The boxed traces represent the length and direction of cell movement of each individually tracked cell plotted from the same point of origin **C.** Box and Whisker graphs show the range of speed and persistence of all tracked control versus PKN knock down cells. The comparison was done using Mathematica software. Stars represent t<0.01 (**) or t<0.5 (*).

To determine the underlying altered property in PKN depleted 5637 cells, we tracked single cell movement during scratch wound assays. Figure 2 shows examples of scratch wounds immediately after wounding (top row) and after 6 h (bottom row), when the untransfected and luciferase transfected controls had closed their wound almost completely, while PKN2 or PKN1/2/3 knock down still showed large open areas. The tracks of individually migrating cells were analysed with Mathematica software and plotted in speed-time graphs (Figure 2B). Control cells reach a maximum speed of 102 µm/hr after 3 h of wounding (median speed 72 µm/hr), while the PKN knock down cells show a rather flat profile over the entire time of the assay (median speed 42 µm/hr). The statistical analysis displayed in the box-and-whisker graphs (Figure 2C) confirms a significant reduction in speed as well as in the persistence of movement in the PKN knock down cells.

### PKN1 and 2 function is necessary for invasion of 5637 bladder tumour cells

**Figure 3:**
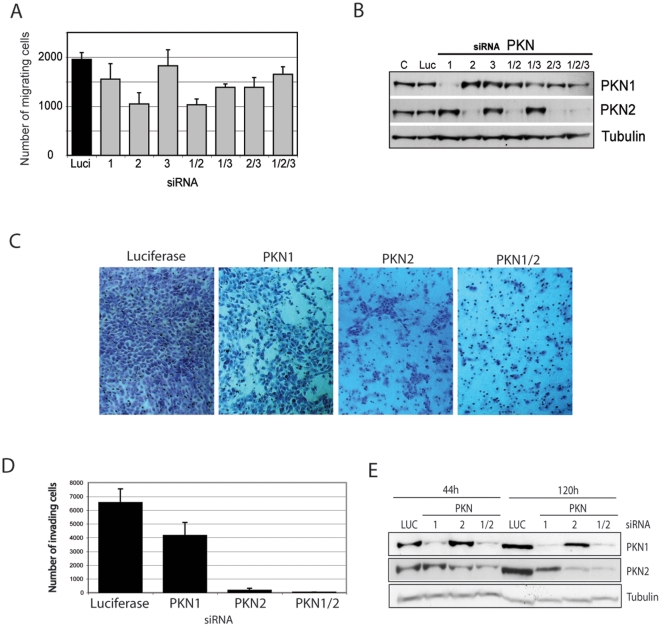
Effect of PKN knock down on tumour cell invasion. **A.** 5637 cells were transfected with siRNA against all PKN isforms either individually or as a double or triple knock down. 48 h post transfection cells were counted and seeded onto laminin coated Transwell membranes. Cells were allowed to migrate along a serum gradient for 12 h. Cells were then fixed at the bottom of the filter and counted. **B.** Western Blot of an equivalent aliquot of the seeded cells confirms the different efficiencies of PKN knock down. **C.** 5637 cells were transfected with siRNA against PKN1or 2 or both. 48 h post transfection cells were counted and then seeded into BD invasion chambers. Invasion was allowed to proceed for 66 h at 37°C. Matrigel was then removed from the inside of the chamber and cells were fixed at the bottom of the chamber and photographed. **D.** Invading cells were counted for each condition (panel C) and compared to the number of invading luciferase control cells. **E.** Equivalent aliquots of PKN depleted cells as seeded into invasion chambers, were taken for western blots as well as for reseeding into 12 well plates and growth during the duration of the invasion assay. This allowed a reference point for PKN knock down after 120 h and confirms that the effect of PKN knock down on invasion is not due to proliferation defects.

It has become clear that cell motility modes and the signalling pathways activated, differ between movement in 2 versus 3 dimensions (see [33]). Therefore, we analyzed whether PKNs were also involved in 3D migration/invasion in this model. 5637 bladder tumour cells showed a similar behaviour in the Transwell migration assay as in the 2D scratch wound assay migrating most efficiently on collagen coated Transwells. Depletion of PKN2 alone in these cells was sufficient to reduce the number of migrating cells by 50% (Figure 3), while depletion of PKN1 resulted in only a 25% reduction when compared to Luciferase knock down cells. As observed in the wound healing assay, in the combination knock-downs and despite some efficiency variation associated with use of combined siRNAs, it is evident that the dominant effect was for PKN2 knock-down.

We next compared 5637 cells for their ability to invade reconstituted basement membrane in the presence or absence of individual PKN isoforms using BioCoat Matrigel invasion chambers. Figure 3C shows images of fixed 5637 cells under control, PKN1, PKN2 and combined PKN1/2 knock-down conditions. Depletion of PKN1 reduced the number of invading cells by 40%, while depletion of PKN2 or both PKN1/2 almost completely abolished invasion (Figure 3D). Interestingly, knock-down of all 3 PKN isoforms partially inhibits invasion of MDA-MB-468 (Figure S2), but depletion of individual PKN isoforms had no effect (data not shown), indicative of PKN redundancy or redundant pathways.

To ensure that the reduction in the number of invading cells is not due to a general defect in 5637 cell proliferation, equal numbers of cells (5×10^4^) were seeded into 12 well plates and kept in culture until the invasion assay was stopped after 66 h. These cells together with an aliquot of cells which was taken at the day of seeding were then processed by western blotting to assess protein expression and knock-down efficiency at the beginning and at the end of the invasion assay (Figure 3). The consistent tubulin levels reflect no effect on division under these conditions, despite the PKN knock-down.

### The PKN2 regulatory domain is required for PKN2 migratory functions in 5637 cells

**Figure 4:**
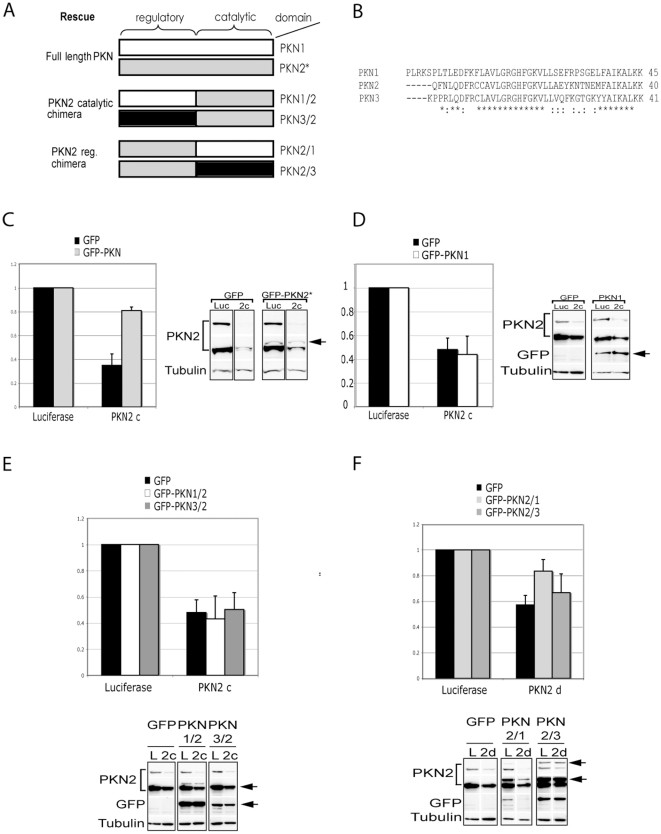
Overexpression of full length PKN2 or a PKN2/1 chimera can rescue migration defects of 5637 cells with PKN2 knock down. **A.** Scheme of siRNA resistant rescue constructs. **B.** Comparison of the initial 40 amino acids of the PKNs catalytic domains used for cloning combinations of PKN regulatory and catalytic domain chimeric constructs. **C.** 5637 cells were stably transfected with GFP or GFP-PKN2* resistant against siRNA 2–3. Both cell lines were grown on 20 µg/ml Collagen and transfected with siRNA against PKN2. 3 days post transfection confluent monolayers were scratched with a pipette tip and wound closure was filmed and analysed using Metamorph software. Cells were then analyzed by western blot for the efficiency of PKN2 knock down and expression of GFP-constructs shown as a short (top panel) and long (bottom panel) exposure. **D.** 5637 stable cell lines expressing GFP or GFP-PKN1 were grown on 20 µg/ml Collagen coated wells, depleted of PKN2 and wounded 3 days post transfection. Wound Healing was monitored over 8 h and analysed with Metamorph software. Western blot analysis was carried out to assess PKN2 knock down efficiency and overexpression of PKN1. **E.** Stable 5637 cell lines expressing GFP, GFP-PKN1/2 or GFP-PKN3/2 chimera were depleted of PKN2 and subject to wound healing assay. Western blot analysis confirms the knock down efficiency of PKN2 and overexpression of GFP-PKN1/2 and GFP-PKN3/2. **F.** 5637 cells overexpressing GFP, GFP-PKN2/1 or GFP-PKN2/3 were transfected with siRNA against PKN2. 72 h post transfection cell monolayers were scratched and wound healing analysed as described above. After 8 h of migration cells were lysed and subject to western blot analysis to determine knock down efficiency and expression levels of PKN chimeras.

To define the redundancy of PKNs we rescued PKN2 knock-down in 5637 cells with different PKN constructs (Figure 4A,B). An siRNA resistant PKN2 construct (PKN2*) stably reintroduced into 5637 cells was able to rescue much of the effect of endogenous PKN2 knock-down in these cells (Figure 4C); 65% inhibition of migration was reduced to ∼20% inhibition on GFP-PKN2* expression. Rescue with the ectopic expression of an siRNA-resistant PKN2 confirms the specificity of the observed effects of PKN2 knock-down. Notably, when GFP-PKN1 was stably expressed there was no rescue of PKN2 depletion in these cells (Figure 4D). This suggests non-redundant functions of these isoforms in migration.

To define further the nature of the non-redundant behaviour, we employed polyclonal, stable, 5637 cell derivatives, expressing chimeric constructs comprising various combinations of PKN isoform regulatory and catalytic domains (Figure 4A,B). Substitution of the regulatory domain of PKN2 with either that from PKN1 or PKN3 compromised the ability of the expressed construct to rescue the effect of PKN2 knock-down (Figure 4E). By contrast expression of the regulatory domain of PKN2 in combination with the catalytic domain of PKN1 partially rescued the effect of PKN2 knock-down. A somewhat variable rescue with the PKN2/3 chimeric construct was observed despite the good levels of expression.

## Discussion

In view of the heterogeneity of PKN isoform expression, to dissect the relative potential of isoforms to control migratory behaviour we screened 22 cell lines for their patterns of PKN isoform expression to establish a suitable model. All cell lines express at least one isoform and the relative levels of individual PKNs showed considerable variation in particular for PKN3. In the tumour cell line 5637 with relatively enriched expression of PKN2 compared to other cells tested, we were able to detect a strong phenotype just by depleting PKN2, with PKN1 depletion having a weaker effect and PKN3 none at all. The results show that unlike the situation reported in prostate cancer cells [18], PKN1 and PKN2 can contribute both to cell migration in wound healing assays as well as 3D invasion of tumour cells in transwells. Thus the PKN isoform contribution to cell motility in part reflects their expression in particular cell systems.

Using the 5637 cell model which is enriched for PKN2 expression, rescue of endogenous PKN2 depletion with the ectopic expression of an siRNA resistant PKN2 confirmed the specificity of the observed effects of knock down. Importantly, this phenotype could not be rescued by simple overexpression of PKN1 or 3. Since we had concluded that in principle any one of these PKN isoforms can contribute to migratory behaviour (albeit in other cell types), this inability to rescue with a heterologous isoform suggested that specific regulatory inputs might distinguish their action. To test this, we constructed a series of chimeric PKN molecules and derived stable cell lines for which we could assess the migratory behaviour ±endogenous PKN2 knock-down. These studies indicated that the regulatory domain of PKN2 was critical for rescue of migration in 5637 cells. Thus, besides full length PKN2 only the chimera PKN2/1 was able to rescue the migration defect on PKN2 depletion in these bladder cells; this was despite the expression of higher levels of other chimeras. The selective ability of PKN2 to rescue over PKN1, and of the PKN2/1 chimera to rescue compared to others, is indicative of specific regulatory inputs acting through the PKN2 regulatory domain to effect its action in migration in this cell model.

The evidence here indicates that although expression patterns seem to reflect the ability of PKN isoforms to influence migration, the patterns themselves are overlaid by the exertion of regulatory inputs that display specificity – in 5637 cells these inputs are principally PKN2 directed. This heterogeneity of regulation is consistent with the findings that there are distinct lipid and Rho family GTPase inputs to this family of kinases. The kinase activity of PKN1 for example is highly stimulated by phosphatidylinositol(4,5)bisphosphate, phosphatidylinositol(3,4,5)trisphosphate, lysophosphatidic acid and arachidonic acid [10], [12]. PKN2 as well as PKN3 are less sensitive to arachidonic acid than PKN1 [14], [34]. With respect to GTPases, PKN1 was shown to bind Rac and RhoA [5] where the HR1a motif binds both Rac and RhoA with high affinity and the HR1b motif binds Rac with much higher affinity than RhoA [35], [36]. PKN1 HR1b is also able to bind RhoA GDP [5]. PKN2 binds and responds to Rac as does PKN1, but the regulation of the kinase by Rho GTPases is still controversial. Quilliam and colleagues [20] found that RhoA binds PKN2 in a GTP dependent manner, but Vincent and Settleman reported that PKN2 binds Rho GDP and Rho GTP with similar kinetics [4]. The extreme C-terminus of PKN2 is also implicated in Rho GTP binding [37], although this specific amino acid sequence seems dispensible for our rescue experiments, since the PKN2/1 chimera will partially rescue. PKN2 and PKN3 contain a proline rich region before the catalytic domain and binding of adaptor proteins known to interact with PKN2 such as Nck or Grb4 has been mapped to this proline rich region [20], [38]. These interactions are not conserved with PKN1. These distinctions in upstream regulators are consistent with the conclusion drawn here that cell-specific regulatory domain inputs determine PKN action in migration.

The single orthologue of the mammalian PKN proteins has been shown to be essential for the development of the Drosophila embryo. Drosophila PKN^-/-^ are embryonic lethal due to defects in dorsal closure [39], a process which involves migration of the lateral epidermal flanks and dynamic changes in cell shape to close a hole in the dorsal epidermis occupied by the amnioserosa. The dorsal closure process has been linked to migration of epithelial cells in other organisms and in a similar manner Rho GTPases have been shown to play an important role [40]. Resonating with the results described in this paper, Betson & Settlement who analyzed the ability of PKN mutants to rescue the dorsal closure defect in Drosophila also found a requirement for the N-terminal domain of PKN in particular to respond to Rho signalling [41]. Interestingly, the kinase domain of PKN could be replaced by that of Protein Kinase C53E which is similar to mammalian PKCα [41]; as observed here, these related AGC kinase domains seem more interchangeable than their regulatory domains.

Migration of 5637 bladder cells was examined previously by Koivunen and colleagues, who demonstrated that cell migration of this epithelial cell line was strongly inhibited by the application of the conventional PKC inhibitor Gö6976. This inhibitor caused translocation of β1 and β4 integrins as well as an increased number of adherens junctions and desmosomes. Together with the data from [41] these findings suggest that PKN and PKCα/β might be linked to the same signalling cascade controlling cell migration.

Migration of cells can occur through individual cell movement in a manner often referred to as amoeboid migration or in a more extended morphology, as well as in a form of collective migration; different requirements are associated with distinct movement behaviours [42]. Collective migration provides the active and passive translocation of mobile and non-mobile cells due to groups of cells being held together by cell adhesion molecules such as E-cadherin. Interestingly, PKN2 has been implicated in the regulation of cell-cell adhesion [43]. Upon Rho stimulation PKN2 either directly or indirectly activates Fyn tyrosine kinase, which in turn phosphorylates β-catenin that binds to E-cadherin and leads to its translocation to cell-cell contacts and increased cell adhesion. The bladder tumour 5637 cells migrate in sheets in the wound assays employed here. It is conceivable that depletion of PKN2 could cause disturbances in cell-cell adhesion and hence slow down migration of these cells in wound healing assays. In adhesion assays of PKN1 and PKN2 depleted cells on different substrates such as collagen and fibronectin, we found that the ability and number of cells adhering to these substrates was no different to luciferase siRNA control cells (data not shown). However, by using immunoflourescene, we noted that focal adhesions seemed larger in cells lacking PKN1 or PKN2 than in control cells (unpublished). Knock-down of the PKN1 downstream target phospholipase D in HeLa cells, also results in increased focal adhesions [44]. Furthermore, 5637 cells when stimulated with proepithelin have been shown to migrate through engagement of a paxillin, FAK and ERK pathway [45]. PKN1 was shown to bind the actin bundling protein α-actinin in a phosphatidylinositol 4,5bisphosphate dependent manner [24]. α-actinin helps to shape dorsal and ventral stress fibers which are anchored with one or both ends in focal adhesions [46]. Disruption of the PKN-actinin interaction could result in defective focal adhesions and impair cell motility. PKN isoforms are also implicated as upstream kinases of mitogen activated kinases such as p38 ([30], [31], [47]). It is possible therefore, that cells depleted of PKN1 and PKN2 have modified focal adhesions that make them adhere stronger to the substrate and might result in a delayed detraction of the cell body during cell migration.

In conclusion, PKN3 is not alone in the PKN family in playing a role in migration. In 5637 cells PKN2 depletion alone inhibits 2D and 3D migration. As the case grows for PKN isoforms as targets for intervention in invasive disease, consideration will need to be given to targeting multiple isoforms in some if not all situations.

## Materials and Methods

### Reagents

Collagen (from calf skin), Fibronectin (from bovine plasma), Laminin (from human placenta) and Fetal Calf Serum were purchased from Sigma-Aldrich. Transwell inserts with 8 µm pores were from Corning and BD Biocoat Matrigel Invasion chambers were obtained from BD Biosciences. PKN1 (also known as PRK1) and PKN2 (PRK2) antibodies were from BD Transduction Laboratories, the tubulin antibody was from Sigma-Aldrich and the actin antibody was from ICN. The GFP antibody was from CRUK monoclonal antibody services.

### Generation of plasmids and site directed mutagenesis

GFP-PKN1 and GFP-PKN2 were as described previously [48].

Polymerase chain reactions were performed using a DNA Engine DYAD (MJ Research). The reaction mix contained: 1U Pfu Turbo, 1x Pfu buffer, 0.4 mM ultrapure deoxynucleotide triphosphate mix (0.4 mM of each dNTP), 5% DMSO, 1 pmol sense primer, 1 pmol antisense primer and 100 ng DNA template. Reactions were made up to 50 µl in distilled water. Primary PCR for PKN regulatory and catalytic domain products, respectively, were performed with one vector primer. Forward (for) and reverse (rev) primers were as follows:

EGFP-for: TCACTCTCGGCATGGACGAG or EGFP-rev: TAAACAAGTTAACAACAACAATTGC and one of the following cDNA specific primers:

pkn1pkn2_cat for

CGCCCTGTGCAGCCCTCTGAGGAAGTCACAGTTTAATCTACAAGATTTCAGG

pkn1pkn2_reg rev

GGAACTTGAAATCTTCGAGGGTCAGAGGAAACCTTTGCTGAGATCTTCTGT

pkn2pkn1_cat for

GAGGACAGAAGATCTCAGCAAAGGTTTCCTCTGACCCTCGAAGATTTC

pkn2pkn1_reg rev

CAACACCTGAAATCTTGTAGATTAAACTGTGACTTCCTCAGAGGGCTGC

pkn2pkn3_cat for

GAGGACAGAAGATCTCAGCAAAGGTTTAAACCCCCTCGGCTTCAGGA

pkn2pkn3_reg rev

CAACACCTGAAATCTTGTAGATTAAACTGCCTGGTGGGGGAGGCTGG

pkn3pkn2_cat for

CCATCTCCACCAGCCTCCCCCACCAGGCAGTTTAATCTACAAGATTTCAGG

pkn3pkn2_reg rev

GCGGAAGTCCTGAAGCCGAGGGGGTTTAAACCTTTGCTGAGATCTTCTGT

PKN2*oligo2–3resistent for

CAACGGCA**C**GG**A**ATGTG**C**CT**T**TATTTGGAACCACAGGGTACT

PKN2*oligo2–3resistent rev

TTCCAAATAAAGGCACATTCCGTGCCGTTGGTTGTCTAAAAAATC

Reactions were conducted with a denaturing step at 95°C, annealing temperature of 62°C and elongation temperature of 72°C for 2 min 20 sec. PCR products were examined by agarose gel electrophoresis and purified using the QIAquick gel extraction kit (Qiagen). Specific regulatory and catalytic PCR products were then pooled to serve as the new template for a second round of PCR amplification in order to generate the desired PKN full-length chimaeras using only primers EGFP-for and EGFP-rev. Reactions were conducted as described above but with an elongation time of 4 min. The total number of cycles for each PCR reaction was 25. PCR products were sequenced verified.

### Cell culture, transfection and generation of stable cell lines

MDA-MB-468, MDA-MB-231, MCF7, A549, Calu-1, HI299, PC3, LNCAP, COLO-741, HT29, COLO320HSR, HEPG2, HeLa, HT1197, HT1376, UMUC3 and 5637 cells were from CRUK cell services. 786-0, A-498, Caki-1, SK-HEP-1 and ACHN transformed human cell lines were obtained from the ATCC. Cells were seeded in 10 or 15 cm dishes for expression studies by Western Blotting and in 24well plates for migration assays. Plasmid transfection was performed with Lipofectamine 2000 (Invitrogen) in Optimem (Life Technologies) according to the manufacturers instructions.

For the selection of stable polyclonal cell lines expressing GFP-PKN chimeras, 5637 cells were split 1∶4 into DMEM in the presence of 500 µg/ml G418 (Sigma) at 36 h post transfection. Resistant cells were then sorted using a FACS sterile sort to separate GFP-positive cells and these were maintained in culture in selection medium.

### Small interfering RNA

HiPerFect reagent was used to achieve knockdown of PKN isoforms using targeted siRNA oligos. Complexes were prepared in DMEM in the absence of serum and oligos targeting PKN isoforms were used at 5 nM each. An oligo targeting luciferase was used as a control in all experiments and also to balance the concentrations of oligos used so that in all cases the final concentration of oligo was 15 nM for MDA-MB-468 cells and 10 nM for 5637 cells. All assays were carried out 48–72 hrs post-transfection. The following target DNA sequences for gene specific siRNAs (Qiagen) were chosen: *LUC* control: AATCGAAGTATTCCGCGTACG; *PKN1a*: AAGGGCACGGGAACTGGAGTT; *PKN1b*: AACTGGAGTTGGCTGTGTTCT; PKN1 (cat56): GAAGGTGCTCCTCTCCGAATT; *PKN2 (oligo2*–*3):* AAGCATGGCATGTGTCTCTATT; PKN2 (cat2): GGATATGGAGATAGAACAA; *PKN3 (GB3)* as described in Leenders et al 2004: GAGAGCCTGTACTGCGAGAAG; *PKN3 (cat3):* CCTGAAGATCGCAGACTTT +

### Western blot analysis

Cells were scraped off culture dishes, lysed in RIPA buffer at 4°C and protein concentration was measured by Bradford [49]. Cell lysates with equal concentrations of protein were then prepared in NuPAGE LDS sample buffer with 0.2 M DTT and heated at 90°C for 10 mins. Samples were run on precast NuPAGE 4–12% gels (Invitrogen) with MOPS SDS Running Buffer containing NuPAGE Antioxidant. RPN800 Full Range Rainbow Markers (Amersham) were run alongside each set of samples. Gels were transferred onto polyvinylidene difluoride membrane (PVDF) or Nitrocellulose membrane (Schleicher & Schuell) using a wet transfer system (Biorad). Membranes were blocked by incubation at room temperature for 1 hour in 3% (v/v) BSA/TBS-T or 5% milk/PBS and then incubated overnight at 4°C in primary antibody diluted 1∶1000. Membranes were washed in TBS-T followed by incubation with the HRP-conjugated secondary antibodies donkey-anti-mouse (Amersham). Membranes were washed again in TBS-T, developed using enhanced chemiluminesence (ECL, Amersham) and exposed to Hyperfilm. For quantitation an ImageQuant LAS 4000 mini instrument was employed to obtain a series of exposures of the ECL readout and ImageJ used to determine the quantitation relative to recombinant PKN proteins.

### Migration assays

Scratch wound assays were performed 72 hrs post-transfection with siRNA oligos on confluent monolayer of cells. A single vertical wound was made in each well with a Gilson D200 pipette tip, medium was replaced with DMEM +10% FCS and where live filming was performed the medium also contained 25 mM Hepes pH 7.2. Plates were sealed around the edge and a small hole made in the side to allow a needle to be inserted through which CO_2_ was blown. A lowlight microscope with moving stage and heating box was used to follow the wound closure over 24 hrs. A 5x phase objective was used and an image taken every 15 mins. After the acquisition had finished the cells were harvested by removing the media, washing once with warm PBS and then adding 50 µl 2 x LDS sample buffer to each well and western blots were performed to confirm successful knockdown of PKN isoforms. The pictures were analysed with MetaMorph software to calculate the area of each wound at each time point up to the point of complete closure of the wound. These values were used to give a speed of migration for each transfection condition and an average of the replicates (at least three) was calculated.

For Boyden chamber migration assays, the undersides of Transwell inserts were coated with either 10 µg/ml laminin, 25 µg/ml fibronectin or 50 µg/ml collagen. These coatings were performed overnight at 4°C. Membranes were then washed, blocked in 0.1% BSA for 1 hr at RT, washed again with PBS and placed into fresh wells of a 24 well plate. 72 hrs post-transfection with siRNA oligos, cells were harvested by trypsin treatment and resuspended in DMEM +10%FCS. The number of viable cells for each sample was counted using a Beckman Coulter Counter. An equal number of viable cells from each transfection condition were pelleted by centrifugation at 1000 g for 5 mins and gently resuspended in DMEM +1% FCS to a final concentration of 4×10^4^ cells/ml. 500 µl of DMEM +10%FCS was placed in the bottom of each well of the plate and 500 µl of cell suspension was added to the upper chamber of each Transwell insert, another sample of cells was taken to analyse the efficiency of knockdown by western blot. The plates were incubated at 37°C, 5% CO_2_ for 5 hrs (MDA-MB-468) or 8 hrs (5637 cells). Thereafter cells remaining in the upper chamber of the Transwell insert were removed by gently cleaning the upper surface of the membrane with a damp cotton bud and washing once in PBS. Cells on the lower surface of the Transwell membrane were fixed in 4% PFA. Cells were permeabilised with 0.1% TritonX-100 in PBS for 10 mins, stained with DAPI (1∶10000 dilution) for 5 min and inserts were washed x 3 with PBS. The extent of cell migration was calculated by counting the number of cells on the lower surface of the membrane. This was achieved using a Discovery microscope by taking nine images of each membrane, which were analysed using Metamorph software and journals to count the number of nuclei in each field

### Invasion assays

DMEM (0.5 ml 37°C) was added to each BD BioCoat Growth Factor Reduced MATRIGEL Invasion Chamber and the matrix was allowed to rehydrate for 2 hrs at 37°C, 5% CO_2_. After rehydration 250 µl of the DMEM was carefully removed and 750 µl of DMEM +10%FCS was placed in the bottom of each well of the plate. Cells were harvested with trypsin 48 hrs post-transfection with siRNA oligos and resuspended in DMEM +10%FCS. The number of viable cells for each sample was counted using a Beckman Coulter Counter. An equal number of viable cells from each transfection condition was pelleted by centrifugation at 1000 g for 5 mins. The cell pellet was gently resuspended in DMEM +1% FCS to a final concentration of 4×10^4^ cells/ml (MDA-MB-468) or 1×10^5^ cells/ml (5637) and 500 µl of cell suspension were added to the upper chamber of each Boyden chamber. Samples of cells were also taken to analyse the efficiency of knockdown by western blot and for seeding into 12 well plates to observe viability of cells. The plates were incubated at 37°C, 5% CO_2_ for 24 hrs (MDA-MB-468) or 64 hrs (5637). Thereafter cells and the matrix in the upper chamber of the Boyden chamber were removed by gently cleaning the upper surface with a damp cotton bud. The inserts were processed and the number of cells that had invaded were counted as for the Boyden chamber migration assays.

## Supporting Information

Figure S1 **Effect of PKN1- 3 knock down on wound healing of MDA-MB-468 cells.** **A.** MDA-MB-468 cells were grown in uncoated 24well plates and depleted of PKN1, 2 and 3 by triple siRNA transfection. 72 h post transfection confluent monolayers were scratched with a pipette tip and wound healing monitored using a lowlight Microscope and analysed by Metamorph software. siRNA (matched concentration) against Luciferase served as control. **B.** 24well plates were coated with 10 µg/ml laminin, 25 µg/ml fibronectin or 50 µg/ml collagen for 1 h at 37°C or overnight at 4°C and blocked with 2% BSA for 30 min at 37°C. MDA-MB-468 cells were then seeded onto the coated multiwell plates, PKN expression was knock-down by siRNA transfection and cells were subjected to a wound healing assay 72 h post transfection.
(TIF)

Figure S2 **Knock-down of PKN1-3 inhibits invasion of MDA-MB-468 cells.** **A.** MDA-MB-468 cells were transfected with siRNA against PKN1/2/3. 48 h post transfection cells were counted and 20 000 cells were seeded into BD invasion chambers. Invasion was allowed to proceed for 48 h at 37°C. Matrigel was then removed from the inside of the chamber and cells were fixed at the bottom of the chamber and photographed. Invading cells were counted for each condition and compared to the number of invading luciferase control cells. **B.** Equivalent aliquots of PKN depleted MDA-MB-468 cells as seeded into invasion chambers, were taken for western blots as illustrated.
(TIF)
