# Supplementary figures and images for: Regulatory Domain Selectivity in the Cell-Type Specific PKN-Dependence of Cell Migration
Source: PLoS One. 2011 Jul 6;6(7):e21732. doi: 10.1371/journal.pone.0021732 (PMC3130767; doi:10.1371/journal.pone.0021732)

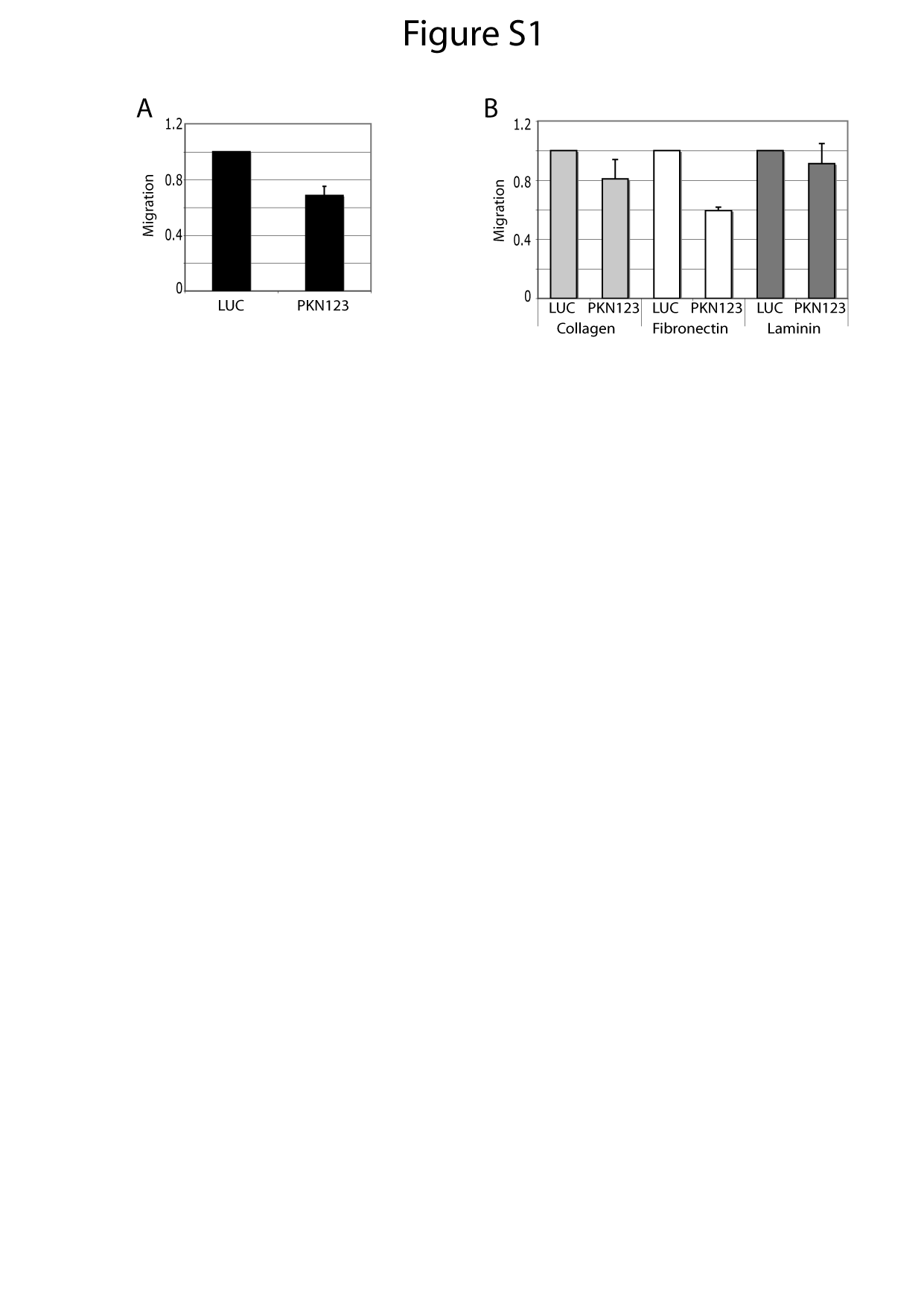

Supplement: Figure S1 — Effect of PKN1- 3 knock down on wound healing of MDA-MB-468 cells. A. MDA-MB-468 cells were grown in uncoated 24well plates and depleted of PKN1, 2 and 3 by triple siRNA transfection. 72 h post transfection confluent monolayers were scratched with a pipette tip and wound healing monitored using a lowlight Microscope and analysed by Metamorph software. siRNA (matched concentration) against Luciferase served as control. B. 24well plates were coated with 10 µg/ml laminin, 25 µg/ml fibronectin or 50 µg/ml collagen for 1 h at 37°C or overnight at 4°C and blocked with 2% BSA for 30 min at 37°C. MDA-MB-468 cells were then seeded onto the coated multiwell plates, PKN expression was knock-down by siRNA transfection and cells were subjected to a wound healing assay 72 h post transfection. (TIF) [file pone.0021732.s001.tif]

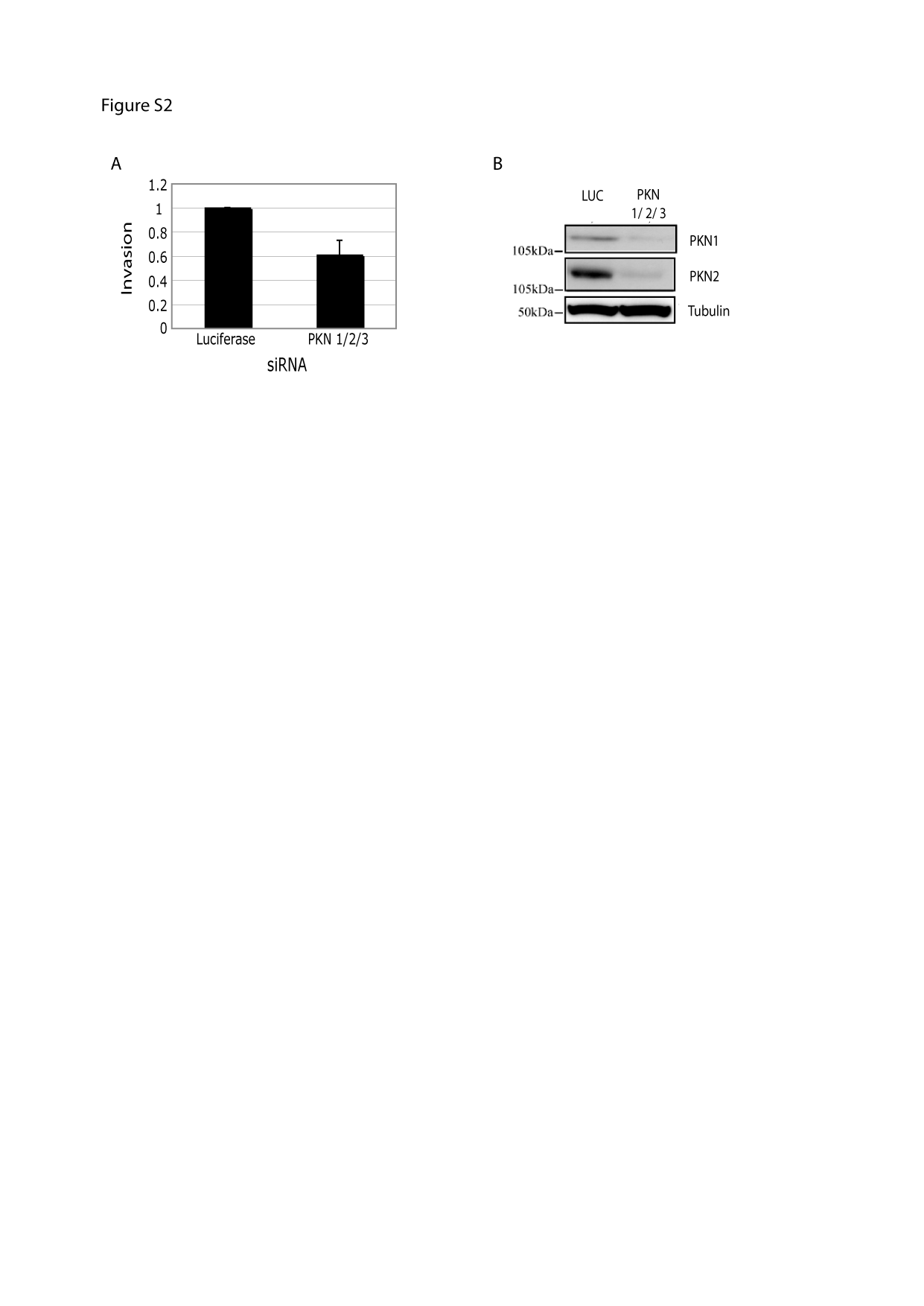

Supplement: Figure S2 — Knock-down of PKN1-3 inhibits invasion of MDA-MB-468 cells. A. MDA-MB-468 cells were transfected with siRNA against PKN1/2/3. 48 h post transfection cells were counted and 20 000 cells were seeded into BD invasion chambers. Invasion was allowed to proceed for 48 h at 37°C. Matrigel was then removed from the inside of the chamber and cells were fixed at the bottom of the chamber and photographed. Invading cells were counted for each condition and compared to the number of invading luciferase control cells. B. Equivalent aliquots of PKN depleted MDA-MB-468 cells as seeded into invasion chambers, were taken for western blots as illustrated. (TIF) [file pone.0021732.s002.tif]
